# HBV X protein mutations affect HBV transcription and association of histone-modifying enzymes with covalently closed circular DNA

**DOI:** 10.1038/s41598-020-57637-z

**Published:** 2020-01-21

**Authors:** Chun Kong Chong, Ching Yan Serene Cheng, Sin Yi Jasmine Tsoi, Fung-Yu Huang, Fen Liu, James Fung, Wai-Kay Seto, Keane K.-Y. Lai, Ching-Lung Lai, Man-Fung Yuen, Danny Ka-Ho Wong

**Affiliations:** 1 Department of Medicine, The University of Hong Kong, Hong Kong, China; 2 State Key Laboratory of Liver Research, The University of Hong Kong, Hong Kong, China; 3 Department of Pathology, City of Hope National Medical Center, Duarte, CA USA; 4 Department of Molecular Medicine, Beckman Research Institute of City of Hope, Duarte, CA USA; 5 City of Hope Comprehensive Cancer, Duarte, CA USA

**Keywords:** Hepatitis B virus, Hepatitis B

## Abstract

The hepatitis B X protein (HBx) plays a role in the epigenetic regulation of hepatitis B virus (HBV) replication. This study investigated the effects of HBx mutations on HBV transcription and the recruitment of HBx, histone acetyl-transferase P300 and histone deacetylase 1 (HDAC1) to circularized HBV DNA (which resembles covalently closed circular DNA [cccDNA]). Compared with wild type, majority of mutants had lower levels of intracellular HBV RNA (44–77% reduction) and secretory HBsAg (25–81% reduction), and 12 mutants had a reduction in intracellular encapsidated HBV DNA (33–64% reduction). Eight mutants with >70% reduction in HBV RNA and/or HBsAg were selected for chromatin immunoprecipitation analysis. Four HBx mutants with mutations in amino acid residues 55–60 and 121–126 had a lower degree of HBx-cccDNA association than wild type HBx (mean % input: 0.02–0.64% vs. 3.08% in wild type). A reduced association between cccDNA and P300 (mean % input: 0.69–1.81% vs. 3.48% in wild type) and an augmented association with HDAC1 (mean % input: 4.01–14.0% vs. 1.53% in wild type) were detected. HBx amino acid residues 55–60 and 121–126 may play an important role in HBV transcription regulation, via their impeded interaction with cccDNA and altered recruitment of histone modifying enzymes to cccDNA.

## Introduction

The hepatitis B virus (HBV) covalently closed circular DNA (cccDNA) is the template for transcription, which produces HBV pregenomic RNA and mRNAs. HBV pregenomic RNA is the template for the generation of the peculiar partially double-stranded, relaxed circular DNA (rcDNA) genome found inside the progeny virions. From the HBV mRNAs, all viral proteins, including the hepatitis B surface antigen (HBsAg), hepatitis B core protein, polymerase, and hepatitis B X protein (HBx), are synthesized. Of these viral proteins, the role of HBx in HBV replication is less well defined.

HBx is a 17 kDa protein with 154 amino acids (aa), consisting of an N-terminal negative regulatory domain (aa 1–50) and a C-terminal trans-activating domain (aa 51–154)^1^. HBx is a promiscuous trans-activator of many cellular genes, and possesses oncogenic properties. Early studies have shown that HBx is required for efficient viral replication^2–5^. However, the mechanism by which HBx facilitates HBV replication has not been well delineated.

cccDNA is associated with cellular histones to form a minichromosome^6^. HBV transcription is regulated by the acetylation status of cccDNA-bound histones^7^. Belloni and colleagues have shown that HBx is associated with the cccDNA minichromosome and other histone modifying enzymes and plays a role in the regulation of HBV transcription^8^. In an HBx-null strain, HBV transcription level is reduced, but can be restored by complementation with an HBx expression plasmid, suggesting that HBx is required for efficient HBV transcription^3^. Another earlier study has identified that the HBx C-terminal trans-activation domain is responsible for efficient HBV replication^2^. However, the details of the HBx subdomains and aa residues involved in HBV transcription have not been fully delineated.

In the present study, we attempted to identify the HBx aa residues that play an important role in HBV replication and its association with cccDNA. The effect of HBx mutations on the recruitment of histone acetyl-transferases and histone deacetylases towards cccDNA was also studied.

## Results

### HBV replication in HBx-negative mutants

We employed an HBx-expression plasmid complement system to assess the effect of HBx mutations on HBV replication. Due to the overlapping nature of the HBV genes, introducing HBx mutations within a full-length HBV DNA genome may alter the coding sequences of the precore/polymerase genes and the Enhancer II sequences. Thus we used an HBx-null full-length HBV DNA (HBxStop) as a background, complemented with either wild type or mutant HBx expression plasmids in our system.

**Figure 1:**
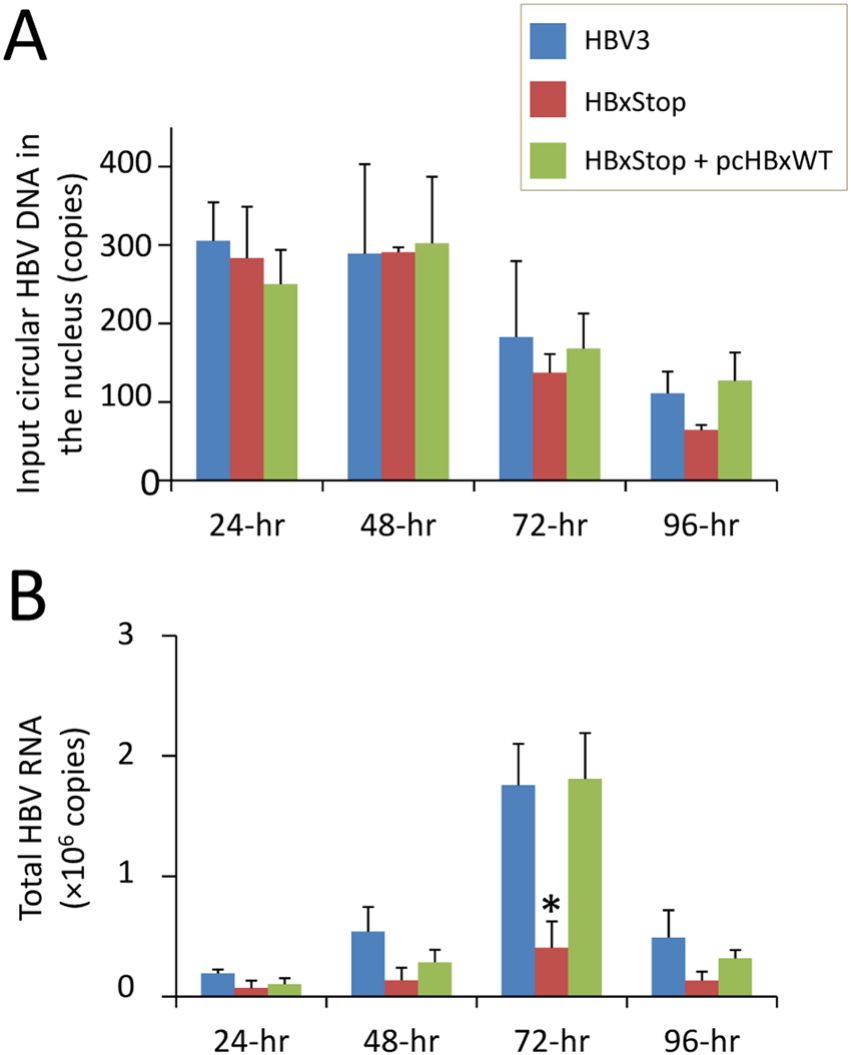
Time course detection of transfected circularized HBV DNA (**A**) and HBV RNA (**B**) following transfection. HBV3: full-length wild type HBV; HBxStop: full-length HBx-negative (stop codon introduced at codon 8) HBV, and HBxStop complemented with wild type HBx plasmid pcHBxWT. Data were obtained based on at least three independent measurements. Asterisks (*) denoted statistically significant differences when compared with HBV3. SEM was denoted by the error bars.

To confirm the role of HBx in HBV replication in our study system, we first compared the replication dynamic of a full-length wild type HBV (named HBV3) and the HBx-negative mutant (HBxStop) with or without complement with HBx expression plasmid (pcHBxWT). The levels of transfected circularized HBV DNA and HBV RNA were assessed at 24, 48, 72 and 96 hours post-transfection (Fig. 1). At all four time points, there was no significant difference in the level of the nucleic circularized HBV DNA in HBV3, pcHBxWT complement strain, and the HBxStop mutant strain, indicating that a comparable amount of the circularized HBV DNA entered and remained in the nucleus. There was a trend of reduction in the level of circularized HBV DNA starting from 48 hours post-transfection. The detectability of HBV RNA at the time points studied, with its peak at 72 hours post-transfection (Fig. 1B), suggesting that this circularized HBV DNA may act as a “cccDNA-like” template for HBV transcription in our system^7,8^. HBxStop mutation resulted in a significant reduction in HBV RNA levels, which were restored to almost wild type level when complement with pcHBxWT. Since the effect of HBx mutations on HBV RNA levels (the focus of this study) was most prominent at 72 hours post-transfection, subsequent analyses were performed at this time point.

### Effect of HBx C-terminal and N-terminal truncation on HBV replication

**Figure 2:**
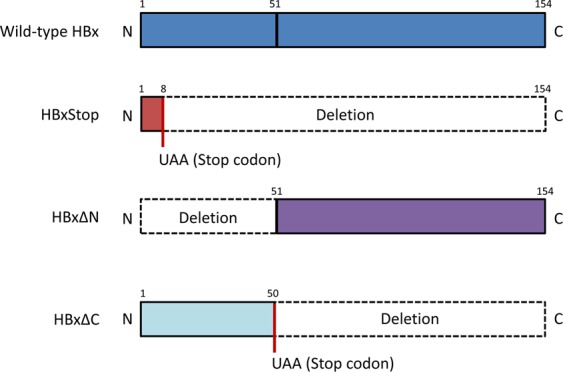
Schematic representation of truncated HBx. Wild type HBx with 154 amino acids in length is schematically shown at the top. HBxStop was created by altering the eighth codon of HBx to an UAA stop codon. Bottom two bars representing the truncated mutant HBxΔN and HBxΔC, with deletions in HBx aa 1 to 50 and 51 to 154, respectively.

**Figure 3:**
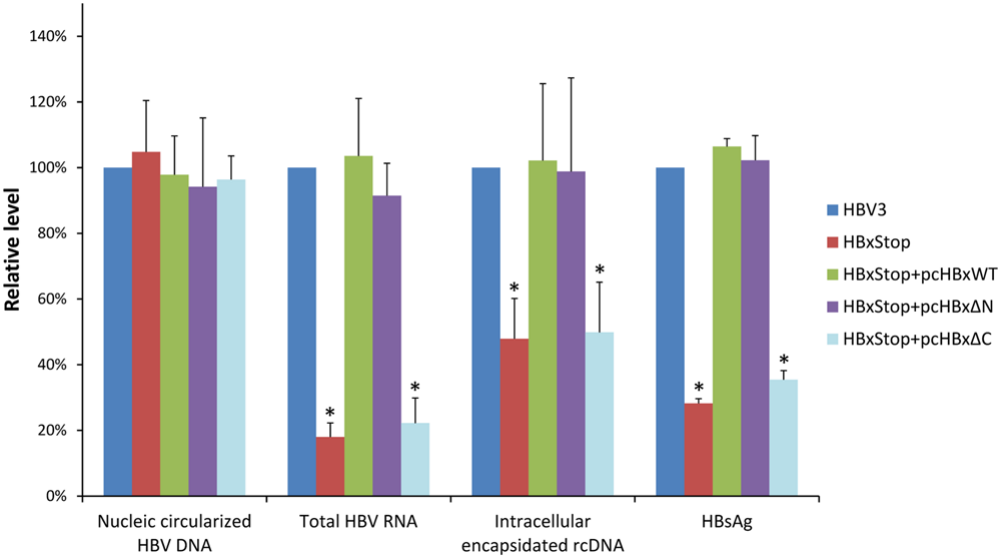
Levels of HBV replication in HBV with wild type HBx and HBx-null or HBx-truncated mutants. The levels of HBV cccDNA, intracellular total HBV RNA, intracellular encapsidated HBV rcDNA, and secretory HBsAg in HBV wild type (HBV3), HBxStop mutant, and HBxStop complemented with wild type HBx, HBxΔN and HBxΔC mutants. Data were obtained based on at least three independent measurements. The Y-axis shows the levels of replication with relative to that of wild type HBV3, which was set to 100% in each set of independent experiment. Asterisks (*) denoted statistically significant differences when compared with the wild type HBV3.

The role of HBx N-terminal and C-terminal domains on HBV replication was then investigated. Two truncated HBx mutant expression plasmids, devoid of HBx N- and C-terminal domains (named HBxΔN and HBxΔC, respectively), were created and schematically illustrated in Fig. 2. HBV replication levels in HBV3, HBxStop, and HBxStop plus HBxWT, HBxΔN or HBxΔC complement strains were measured. There was no significant difference in the level of circularized HBV DNA among the wild type and HBx deletions and truncation mutant strains (Fig. 3). Compared with the wild type HBV3, the HBxStop mutant had a significantly lower level of HBV RNA (18% of wild type), intracellular encapsidated rcDNA (48% of wild type), and secretory HBsAg (28% of wild type). Upon co-transfection of HBxStop with pcHBxWT, the levels of HBV RNA, rcDNA, and HBsAg were restored to a level comparable to wild type HBV3. However, wild type replication levels were restored only by HBx∆N, but not by HBx∆C, suggesting that the HBx C-terminal is crucial in producing a wild type level of HBV replication.

### Mutagenesis of hepatitis B X protein

We further fine-mapped the HBx aa residues by studying the replication characteristics of HBV mutants with point mutations within the HBx C-terminal domain. Using an expression plasmid pcHBxWT as a template, 30 HBx mutants with mutations in the C-terminal domain were created by site-directed mutagenesis. Each mutant was designed by substituting 3 to 7 consecutive aa residues in HBx C-terminal region to alanine residues. A schematic representation of the HBx mutants, named MT1-MT30, is shown in Supplementary Fig. 1.

### HBx protein expression

**Figure 4:**
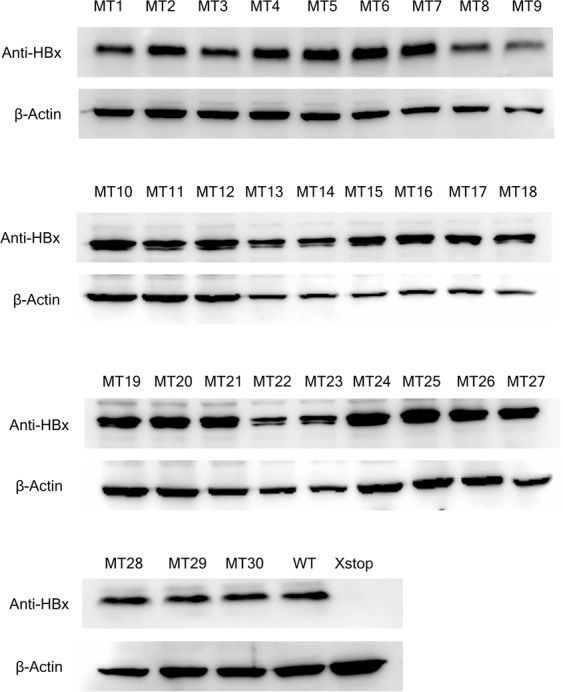
Western blot detection of mutant (MT1-30) and wild type HBx proteins. Detection of β-actin was used as the internal control. WT, pcHBxWT; X-stop, HBxStop null mutant.

Figure 4 shows a representative Western blot analysis of the wild type and mutant HBx. A protein band of approximately 17 kDa was detected in wild type HBx and the 30 HBx mutants but not in HBxStop. Statistical analysis of protein expression levels, based on densitometry scanning of the relative band intensities of HBx and β-actin, showed that there were no significant differences in the expression levels of the wild type and mutant HBx (all *P* > 0.05). This suggested that HBx expression levels were not impaired by the mutations created.

### Effect of HBx alanine mutations on HBV replication

**Figure 5:**
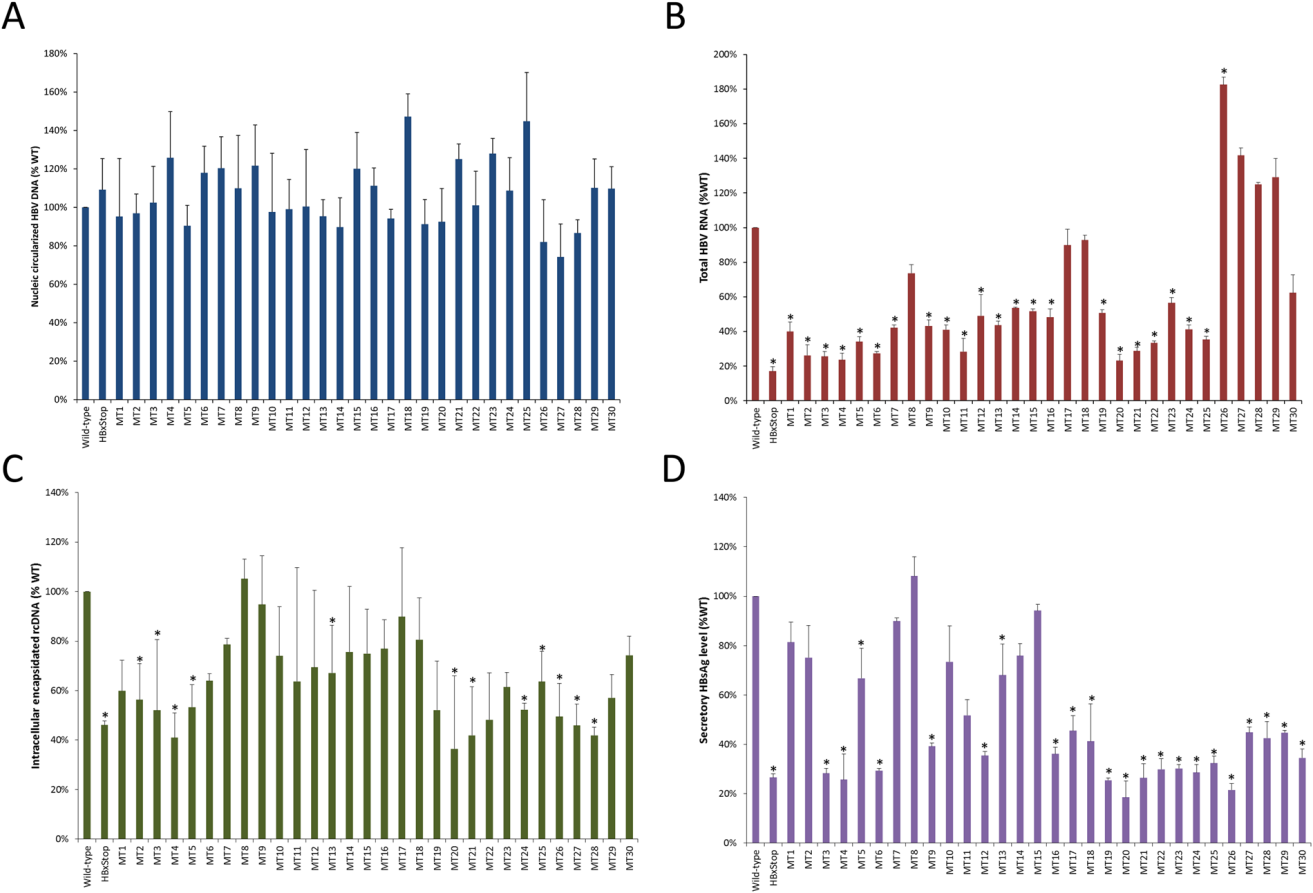
Assessment of HBV replication with 30 HBx mutations (MT1-30). The diagrams representing the relative levels of (**A**) circularized HBV DNA in the nucleus; (**B**) total HBV RNA; (**C**) intracellular encapsidated HBV rcDNA; and (**D**) secretory HBsAg. Data were obtained based on at least three independent measurements. The Y-axis shows the levels of replication with relative to that of wild type HBV3, which was set to 100% in each set of independent experiment. Asterisks (*) denoted statistically significant differences when compared with that of HBxStop complemented with pcHBxWT. SEM was denoted by the error bars.

The effects of the 30 HBx point mutations on HBV replication were assessed using HBxStop complemented with either pcHBxWT or mutant HBx (MT1-30) expression plasmids. As shown in Fig. 5A, the levels of transfected circularized HBV DNA in the nucleus were comparable in all strains (all *P* > 0.05), indicating that similar levels of circularized HBxStop DNA were detected in the nuclei following transfection. Compared with the wild type HBx complement strain, HBxStop had a lower level (17% of wild type) of HBV RNA (*P* < 0.001; Fig. 5B). In general, HBx alanine mutants MT1-25 (aa 51–138; with the exception of MT8, 17, and 18) showed a significantly reduced total HBV RNA levels (44–77% reduction from wild type; all *P* < 0.05). However, the HBV RNA level in mutant MT26 was higher than wild type. The differences in HBV RNA levels between wild type HBx and distal C-terminal HBx mutants (MT27-30) were not statistically significant. These results indicated that HBx aa 51–141 might play a role in HBV transcription.

Figure 5C depicts the level of encapsidated rcDNA in the wild type and mutant HBx strains. HBxStop had a reduced level of encapsidated rcDNA (46% of wild type level, *P* = 0.001). The HBx mutants MT2-5, MT13, MT20-21, and MT24-28 had a significant reduced rcDNA level (33–64% reduction compared with wild type; all *P* < 0.05). The levels of rcDNA in mutant MT1 and MT22 were slightly lower than that of wild type (*P* = 0.057 and 0.052, respectively). In the rest of the HBx mutants, the levels of rcDNA were not significantly different from that of wild type HBx.

The level of production of secretory HBsAg in the culture supernatant was measured (Fig. 5D). Similarly, the HBxStop mutant had a reduced HBsAg level when compared to wild type (*P* < 0.001). A significant reduction in HBsAg levels was also detected in the majority of HBx mutants (MT3-6, MT9, MT11-13 and MT16-30; 25–81% reduction; all *P* ≤ 0.01), while the reduction of HBsAg in the rest of the mutants was not statistically significant.

The above results showed that these 30 HBx mutants affected HBV replication in various ways. In the subsequent analysis, we narrowed down the scope of study by focusing on a smaller number of HBx mutants. As HBV transcription was the focus of this study, we put a heavier weight into the effect of these mutants on HBV RNA levels. We also attempted to select mutants located in dispersed regions within the HBx C-terminal so that the role of different HBx C-terminal regions in its interaction with the circularized DNA, which resemble the cccDNA molecules, could be studied. As shown in Fig. 5B, among the 30 mutants, eight mutants, namely MT2, 3, 4, 6, 11, 20, 21 and 24, had a relatively low level of HBV RNA. In general, the majority of these eight mutants also had a low level of encapsidated rcDNA and HBsAg (Fig. 5C,D). Therefore, these eight HBx mutants, with >70% reduction in HBV RNA and/or HBsAg, were selected for further study. It has been shown in previous studies that this transfected HBV DNA is associated with cellular histone H3 and H4, with HBV replication levels correlated the extent of histone acetylation^7,8^. Therefore, the transfected circularized HBV DNA will hereafter be referred to as “cccDNA”.

### Association of HBx mutants and histone modifying enzymes with cccDNA

**Figure 6:**
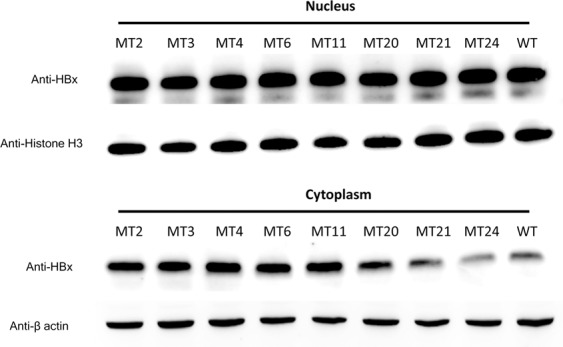
Western Blot detection of HBx in the nuclear and cytoplasmic fractions of the selected mutants. Histone-H3 and β-actin were used as the internal loading control for the nuclear and cytoplasmic fractions, respectively. WT, pcHBxWT.

**Figure 7:**
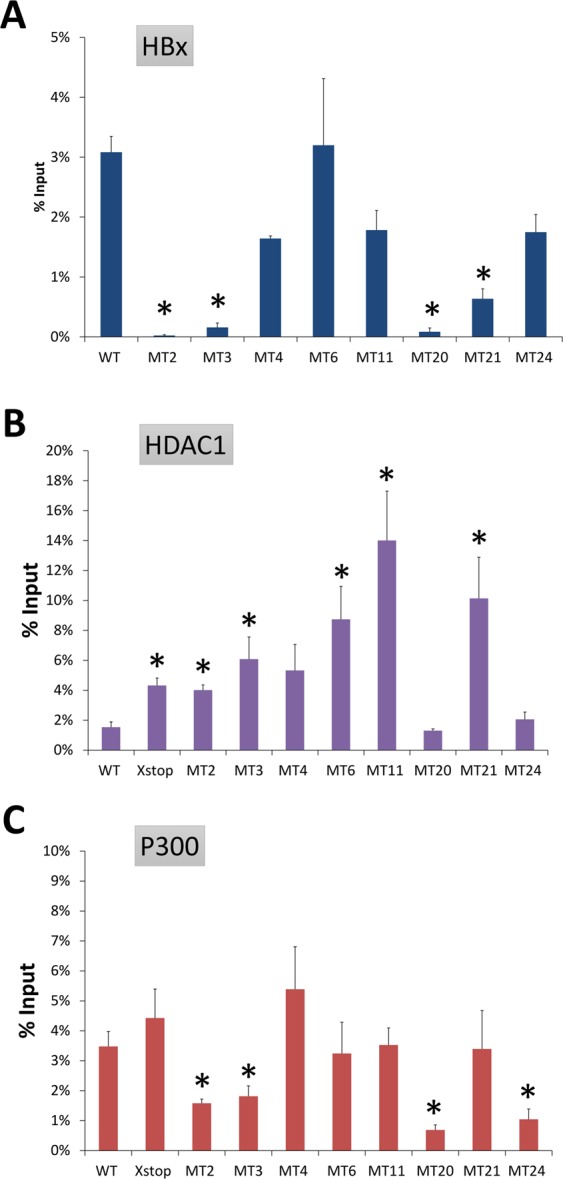
Chromatin Immunoprecipitation (ChIP) analysis of the association of HBx and histone modifying enzymes with HBV cccDNA. The association of HBx (**A**); HDAC1 (**B**); and P300 (**C**) with cccDNA was assessed. The degree of association of proteins of interest with cccDNA was expressed in mean percentage of input (% input). Data were obtained based on at least three independent measurements. Asterisks (*) denoted statistically significant differences when compared with HBxStop complemented with pcHBxWT. SEM was denoted by the error bars. WT, HBxStop complemented with pcHBxWT; X-stop: HBxStop null mutant.

Western Blot analysis of the nuclear and cytoplasmic protein fractions showed that nucleic-cytoplasmic distribution of HBx were not affected in the eight selected HBx mutants studied (Fig. 6). ChIP assay was performed to study whether the HBx mutations affect the association between HBx and circularized cccDNA. Compared with the wild type HBx, mutants MT2-3 and MT20-21 had a significantly lower degree of association with cccDNA (mean % input: 0.02–0.64% vs. 3.08% in wild type; *P* < 0.05; Fig. 7A). The association between cccDNA and the other four HBx mutants (MT4, MT6, MT11, and MT24) were not significantly reduced compared with the wild type HBx.

The association between histone acetyl-transferase P300 and histone deacetylase (HDAC1) with cccDNA was studied. HBxStop mutation caused a significantly enhanced degree of association between cccDNA and HDAC1 (mean % input: 4.32% vs. 1.53% in wild type, *P* = 0.004; Fig. 7B). However, we did not find a significant difference between wild type HBx and HBxStop in the association between P300 and cccDNA (Fig. 7C).

Among the eight selected HBx alanine mutants, the degree of association between P300 and cccDNA was significantly lower than wild type in MT2, MT3, MT20, and MT24 (mean % input: 0.69–1.81% vs. 3.48% in wild type, all *P* < 0.05; Fig. 7C). In contrast, the HBx alanine mutants generally showed an increased association between cccDNA and the histone deacetylase HDAC1. Specifically, an increased recruitment of HDAC1 to cccDNA was detected in MT3, MT6, MT11, and MT21 (mean % input: 4.01–14.0% vs. 1.53% in wild type; *P* < 0.05; Fig. 7B).

**Figure 8:**
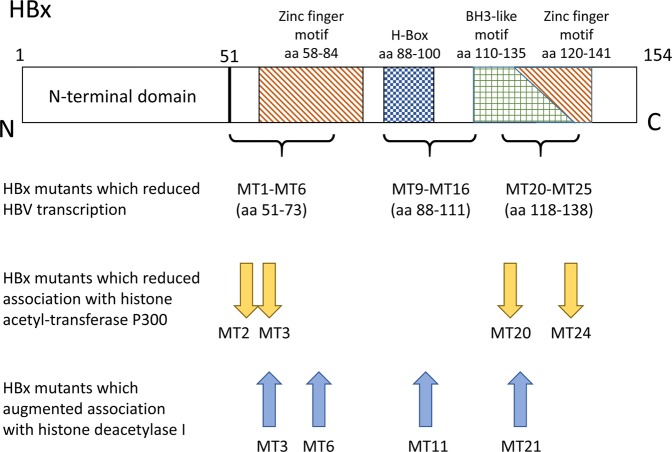
Schematic representation of the HBx protein and the subdomains, as well as the positions of the mutations and the effects. The HBx protein is shown as the long rectangle, with the N terminal on the left and the C terminal on the right. The domains are shown as patterned boxes. There was a 15 amino acid overlap between the BH3-like motif (aa 110–135) and the zinc finger motif (a 120–141). Positions of the mutants studied are listed below the HBx rectangle.

Taken together, these ChIP assay data suggested that the mutant HBx proteins had a decreased association with the circularized HBV DNA, which resembles the cccDNA minichromosome complex in this study. In the strains with HBx alanine mutations, the association between histone acetyl-transferase P300 and cccDNA was reduced, while that between histone deacetylase HDAC1 and cccDNA was enhanced.

## Discussion

In the present study, we used a plasmid-free circularized HBV DNA template so that HBV transcription from endogenous HBV promoters (not from plasmid-borne promoter) can be appropriately studied. This transient transfection system with plasmid-free monomeric HBV DNA has been demonstrated previously to recapitulate the HBV replication cycle from the generation of cccDNA-like transcription templates to the production of progeny viral particles^7,8^. Consistent with previous findings, the present study showed that HBx C-terminal deletion resulted in a decreased HBV replication level^2^.

In the HBx-negative mutant, the association of cccDNA with HDAC1 was enhanced. Although a direct action of HBx to recruit or block histone acetyl-transferases or histone deacetylases towards cccDNA cannot be proven in this study, our result is consistent with previous studies suggesting that HBx takes part in the regulation of HBV transcription via a change in the acetylation status of cccDNA-bound histones^4,7,8^.

The level of HBV replication in cells co-transfected with HBxStop and the 30 HBx alanine mutants was further studied. All these HBx mutant proteins with point mutations were detectable by Western Blot, suggesting the HBx protein stability was not affected by the mutations. Compared with cells transfected with wild type HBx, majority of the HBx point-mutants had a significant reduction in HBV RNA, and secretory HBsAg, while approximately half of the HBx point-mutants had a significant reduction of encapsidated rcDNA.

Based on the extent of the effect of these point-mutants on HBV replication, especially HBV transcription, eight mutants were selected for further investigation. ChIP analysis revealed that four of the eight mutants (MT2, MT3, MT20 and MT21) showing a significant reduction in its association with cccDNA, despite that their nuclear-cytoplasmic distribution was comparable. This indicates that HBx amino acid residues at positions 55–60 and 121–126 are influential to the association of HBx itself to the cccDNA minichromosomal complex.

We also found that four mutants (MT2, MT3, MT20, and MT24) had a significantly reduced association between the histone acetyl-transferase P300 and cccDNA. In addition, four mutants (MT3, MT6, MT11, and MT21) had augmented association between histone deacetylase HDAC1 and cccDNA. In general, these regions of HBx (aa 55–60 in MT2-3, aa 67–73 in MT6, aa 94–96 in MT11, and aa 121–135 in MT20-24) may play a role in facilitating the recruitment of histone acetyl-transferase P300 to cccDNA and/or somehow deterring the recruitment of histone deacetylase to cccDNA. From these ChIP results, we reason that these HBx regions may be important in the recruitment of HBx to cccDNA, thereby affecting the recruitment of histone acetyl-transferase and deacetylase to cccDNA, which alters the acetylation status of cccDNA-bound histones and facilitates HBV transcription.

HBx is a multifunctional protein that may augment HBV transcription through its trans-activating activity and its interaction with other cellular proteins. The HBx C-terminal has two zinc-finger motif-containing trans-activating regions at aa 58–84 and 120–141 (corresponding to MT3-MT7 and MT20-MT26 in this study), as well as other motifs such as the Bcl-2 homology 3 (BH3)-like motif (aa 110–135) and the H-box motif (aa 88–100; covering MT8-MT12) ^9–16^. HBx binds to anti-apoptotic Bcl-2 proteins via the BH3-like motif within HBx (aa 110–135)^17,18^. Mutations within the BH3-like motif at HBx aa 124 and 127 (covering MT21 and MT22) has been reported to prevent its binding to the anti-apoptotic Bcl-2 proteins, thereby abrogating cytosolic calcium elevation, and reducing HBV replication^17,18^. In another study, Guo *et al*. found that the HBx H-box motif regulates HBV replication both positively and negatively through different pathways^14^. It is possible that, in addition to the altered association between cccDNA and histone acetyl-transferases/deacetylases, other transactivating activities of HBx may play a role in HBV transcription. For example, in this study, there was no significant difference between WT and MT4 in the association of cccDNA with P300 and HDAC1, while MT4 caused a significant reduction of intracellular HBV DNA, HBV RNA and HBsAg compared with WT. Thus, the effect of HBx mutations on HBV replication identified in this study may be a consequence of several mechanisms. The positions of the HBx domains, as well as the position of the mutations which affects HBV transcription and the association between cccDNA and P300 and HDAC1, is schematically shown in Fig. 8.

HBx also enhances HBV replication by promoting the degradation of the “structural maintenance of chromosomes” (Smc) complex Smc5/6^19,20^. In the absence of HBx, active Smc5/6 serves as a restriction factor that blocks the transcription from extrachromosomal HBV DNA. In the presence of HBx, Smc5/6 is degraded, thereby allowing HBV transcription. The present mutational analysis of HBx protein may provide an ideal platform for further studies of its domains responsible for Smc5/6 degradation. Further works will be performed to determine whether these HBx mutants affect Smc5/6 degradation.

One limitation of this study concerns the transient transfection system used. Theoretically, a stable HBxStop cell line may produce findings that are more concrete. However, the use of stable cell line will have to employ a >1.1 genome length HBV DNA, with pregenomic RNA expression driven by foreign promoter. Thus the effect of HBx on the HBV transcription may be masked by the activity of the foreign promoter in the stable cell line. For this reason, we employed a transient transfection complement system with circularized HBV DNA carrying the same sequence as cccDNA. Although chromatinization of the transfection circularized HBV DNA has not been experimentally proven in this transient transfection system, this experimental set-up has been demonstrated to recapitulate the HBV replication cycle^7^. In addition, the association between the transfected DNA and cellular histones H3 and H4, as well as the effect of HBV proteins on HBV replication, has been studied in this system^7,8,21^.

The second limitation is that this study could not identified important HBx amino acids down to a single acid level; it identified important HBx domains only at a resolution of 3–7 amino acids. Thirdly, due to the overlapping nature of the HBV RNA, technically, the effect of HBx mutations on individual HBV promoters could not be measured. Given the slight discrepancies between the level of total HBV RNA, intracellular encapsidated rcDNA (which reflects the level of the pregenomic and precore RNA), and secretory HBsAg (which reflects the level of the S mRNAs), we speculate that the effects of HBx on individual HBV promoters are slightly different. Finally, this study only demonstrated the association, but not the causal relationship, between the HBx mutations, HBV transcription, and the interaction between cccDNA with HBx and histone modification enzymes. Based on the previous study that demonstrated the causal relationship between histone acetylation and HBV transcription^7^, our findings of the effect of HBx mutations on the association between histone acetyl transferases/deacetylases with cccDNA likely suggested that HBx mutations had a causal effect on HBV transcription.

The present study identified that HBx aa 55–60 and 121–126 are important in regulating HBV transcription via altering the acetylation status of cccDNA-bound histone. These findings may provide clues to the discovery of inhibitors or antibodies against these functional HBx domains, which may block histone acetylation and suppressing HBV transcription. This treatment strategy may be used in combination with currently approved HBV reverse transcriptase inhibitors or other drugs in research pipelines.

## Methods

### Cell lines and plasmids

A transient transfection system was employed in this study. HepG2 cells expressing the sodium taurocholate cotransporting polypeptide (NTCP) was a kind gift from Professor DY Jin, University of Hong Kong. A full-length HBV DNA was amplified from serum collected from a 31 years old male chronic hepatitis B patient, using primers and methods described previously^22^. Written informed consent was obtained from the subject. This study was approved by the Institutional Review Board of the University of Hong Kong and Hospital Authority, Hong Kong West Cluster. All experiments were performed in accordance with relevant guidelines and regulation.

The HBV amplicon was digested with *Sac*I (New England Biolabs, Ipswich, MA, USA) and cloned into pUC19. Sequence analysis of HBV insert in the clone, named pHBV3, showed that it was of HBV genotype C, without any known drug resistance mutations and basal core promoter and precore mutations. From this plasmid pHBV3, the single genome length HBV DNA was released by *Sap*I (New England Biolabs) digestion, followed by self-ligation to generate circularized HBV DNA. This circularized HBV DNA, named HBV3, was checked by agarose gel electrophoresis (Supplementary Fig. 2).

An expression plasmid containing wild type X gene, named pcHBxWT, was constructed by PCR amplification of the X gene with primers HBx-s (5′-GATC*GGTACC*AATGGCTGCTAGGGTGTGC-3′; *Kpn*I site in italic) and HBx-a (5′-AGCTT*GAATTC*TTGAACAGTAGG-3′; *EcoR*I site in italic), followed by cloning of the amplicon into pcDNA3.1. Similarly, two truncated-HBx plasmids, named HBxΔN (devoid of aa 1–50) and HBxΔC (devoid of aa 51–154) were created by PCR using HBx-delN-s (5′-CCAA*GGTACC*A**ATG**GCGCACCTCTCTTTA-3′; HBx start codon in bold) and HBx-a primer pair and HBx-s and HBx-delC-a (5′-AAAGA*GAATTC***TTA**CCCGTGGTTGG-3′; stop codon in bold) primer pair, respectively.

### Site-directed mutagenesis

Site-directed mutagenesis was performed using the QuikChange Lightning site-directed mutagenesis kit (Agilent Technologies, Santa Clara, CA, USA). To make an HBx-null background, a nonsense mutation at codon 8 of the X gene was introduced into pHBV3. The HBx-null HBV DNA was released by restriction digestion, followed by self-ligation as mentioned above to make circularized HBx-negative HBV DNA (HBxStop). The 30 HBx mutants were created by site-directed mutagenesis using pcHBxWT as template and primer pairs for site-directed mutagenesis (Supplementary Table 1).

### Transfection

Approximately 800 ng of circularized HBxStop DNA was co-transfected with 200 ng of pcDNA3.1, pcHBxWT, or MT1-MT30 into 1 × 10^6^ HepG2-NTCP cells, using Lipofectamine 3000 (Thermo Fisher Scientific, Waltham, MA, USA). A green fluorescent protein expression plasmid was used to monitor the transfection efficiency, which was determined to be around 30–40% (data not shown).

### Extraction of HBV DNA and RNA

Unless otherwise stated, cells were harvested at 72 hours post-transfection. HBV DNA inside the intracellular core capsid particles and nuclei was purified, as previously described^21^. Briefly, harvested cells were lysed in ice-cold homogenization buffer (50 mmol Tris-Cl pH 7.4, 1 mmol EDTA, and 1% Nonidet P-40), which differentially disrupted the plasma membrane without breaking the nuclear membrane. The partial lysate was then subjected to centrifugation at 16,000 × g at 4 °C for 15 minutes to separate the nucleic (pellet) and cytoplasmic (supernatant) fractions, from which the transfected circularized HBV DNA and the encapsidated HBV rcDNA, respectively, were extracted using the Purelink Genomic DNA mini kit (Thermo Fisher Scientific). Prior to the extraction of the encapsidated rcDNA in the cytoplasmic fraction, 250 units of Pierce Universal Nuclease (Thermo Fisher Scientific) were added to the supernatant to eliminate the excess non-encapsidated input HBV DNA in the cytoplasm. We have previously demonstrated that, following nuclease digestion, only negligible amount (0.0001% of input) of non-encapsidated HBV DNA was detected in the cytoplasm^21^, indicating that the nuclease could efficiently eliminate the non-encapsidated transfected DNA in the cytoplasm.

Total cellular RNA was extracted using the TRIzol reagent (Thermo Fisher Scientific) and purified by DNase I treatment. The quantity and quality of DNA and RNA were assessed by the Nanodrop 2000 Spectrometer (Thermo Fisher Scientific).

### Real-time PCR/RT-PCR quantification of HBV DNA/RNA

A previously established real-time PCR method was used to measure HBV DNA and circular HBV DNA (which has the same sequence as cccDNA) in transfected cells^23–26^. rcDNA inside the intracellular capsids was measured using primers spanning the HBV S region (HBV250s: 5′-AGACTCGTGGTGGACTTCTCTCA-3′ and HBV424a: 5′-GAACCAACAAGAAGATGAGG-3′; 0.4 µM each) and the QuantiNova SYBR Green PCR kit (QIAGEN, Hilden, Germany). The level of circularized HBV DNA was measured using the Quantifast Probe PCR kit (QIAGEN), with primers spanning the incomplete region in the HBV rcDNA (HBV1548s: 5′-CTCCCCGTCTGTGCCTTCT-3′ and HBV1886a: 5′-GCCCCAAAGCCACCCAAG-3′; 0.6 µM each), fluorescence probes (5′-GTTCACGGTCTCCATGCAACGT-FAM-3′ [0.2 µM] and 5′-LC_640_-AGGTGAAGCGAAGTGCACACGGACC-phosphoramidite-spacer-3′ [0.4 µM]). The thermal-cycle conditions are described previously^23^. Prior to amplification of the circular DNA, DNA extracted from the nucleus was digested by Plasmid-safe DNase (Epicentre, Madison. WI, USA) to eliminate the cellular DNA and linear HBV DNA. Total HBV RNA was detected using one-step real-time RT-PCR with the Quantifast Probe RT-PCR kit (QIAGEN) and the same primers (Forward: 5′-CTCCCCGTCTGTGCCTTCT-3′ and reverse: 5′-GCCCCAAAGCCACCCAAG-3′) and probes (5′-GTTCACGGTCTCCATGCAACGT-FAM-3′ and 5′-LC_640_-AGGTGAAGCGAAGTGCACACGGACC-phosphoramidite-spacer-3′) as in cccDNA detection^21^. The thermal-cycle conditions were 50 °C for 10 minutes and 95 °C for 5 minutes, followed by 45 cycles of 95 °C 10 seconds, 52 °C for 15 seconds and 72 °C for 15 seconds. Real-time PCR/RT-PCR reactions were performed in the RotorGene Q PCR System (QIAGEN).

### Detection of viral proteins and antigens

HBx was detected using anti-HBx mouse monoclonal antibodies (Abcam, Cambridge, UK) and HRP-conjugated anti-mouse IgG secondary antibodies (Cell Signaling Technology, Danvers, MA, USA) by SDS-PAGE and Western Blot. β-actin and histone H3 were used as the protein loading controls for total cell lysate and nuclear protein fraction, respectively. HBsAg in the culture medium was detected by the Elecsys HBsAg II assay (Roche Diagnostics, Indianapolis, IN, USA).

### Chromatin-immunoprecipitation assays

The MagNA-Pure chromatin immunoprecipitation kit (Thermo Fisher Scientific) was used in ChIP experiments. Briefly, transfected cells were fixed in 1% formaldehyde. The chromatin were sheared by sonication and immunoprecipitated using anti-HBx, anti-P300, and anti-histone deacetylase 1 (HDAC1) antibodies (Abcam). The immunoprecipitated chromosomal DNA and circularized HBV DNA were released by reverse cross-linking and subsequently digested with Plasmid-safe DNase which degraded the linear chromosomal DNA but not the circularized HBV DNA. The ChIPed circularized DNA content was then measured using real-time PCR with the same circularized HBV DNA primers described above. A negative control, using nonspecific immunoglobulin (Cell Signaling Technology) was included. ChIP results were expressed as percentage of input (% input), where input referred to the equivalent amount of an aliquot of the starting cross-linked chromatin collected before the immunoprecipitation step.

### Statistical analysis

All statistical analyses were performed using SPSS21.0 (SPSS Inc, Chicago, IL, USA). Continuous variables were expressed as mean ± standard error of the mean (SEM) and analyzed using the two-tailed Student’s t-test. Data were obtained based on at least three independent measurements. A *P* value < 0.05 denoted statistical significance.

## Supplementary information

Suppementary Materials.

## Data Availability

The datasets generated during and/or analyzed during the current study are available from the corresponding author on reasonable request.
