# Supplementary material for: HBV X protein mutations affect HBV transcription and association of histone-modifying enzymes with covalently closed circular DNA
Source: Sci Rep. 2020 Jan 21;10:802. doi: 10.1038/s41598-020-57637-z (PMC6972884; doi:10.1038/s41598-020-57637-z)

Supplementary Materials

HBV X protein mutations affect HBV transcription and association of histone-modifying enzymes with covalently closed circular DNA

Chun Kong Chong,[1] Ching Yan Serene Cheng,[1] Sin Yi Jasmine Tsoi,[1] Fung-Yu Huang,[1] Fen Liu,[1] James Fung,[1,2] Wai-Kay Seto,[1,2] Keane K-Y Lai,[3,4,5] Ching-Lung Lai,[1,2] Man-Fung Yuen,[1,2] Danny Ka-Ho Wong[1,2]

[1] Department of Medicine, The University of Hong Kong, Hong Kong SAR

[2] State Key Laboratory of Liver Research, The University of Hong Kong, Hong Kong SAR

[3] Department of Pathology, City of Hope National Medical Center, Duarte, CA, USA.

[4] Department of Molecular Medicine, Beckman Research Institute of City of Hope, Duarte, CA, USA

[5] City of Hope Comprehensive Cancer, Duarte, CA, USA

Supplementary Table 1. Primers used for mutagenesis of HBx

Primer name	Primer sequence (5′-3′)	Genome position
HBx-MT1-s	GGCCAACCACGGGGCGGCCGCCGCTTTACGCGGTCTCCC	1527-1566
HBx-MT1-a	GGGAGACCGCGTAAAGCGGCGGCCGCCCCGTGGTTGGCC	1527-1566
HBx-MT2-s	CCAACCACGGGGCGCACCTCTCTGCAGCCGCTCTCCCCGTCTG	1536-1544
HBx-MT2-a	CAGACGGGGAGAGCGGCTGCAGAGAGGTGCGCCCCGTGGTTGG	1536-1544
HBx-MT3-s	TTACGCGGTGCCGCCGCCTGTGCCTTCTCAT	1545-1576
HBx-MT3-a	ATGAGAAGGCACGCGGCGGCGCACCGCGTAA	1545-1576
HBx-MT4-s	CCCGTCGCTGCCGCCTCATCTGCCGGACC	1554-1583
HBx-MT4-a	GGTCCGGCAGATGAGGCGGCAGCGACGGG	1554-1583
HBx-MT5-s	CGTCTGTGCCTTCGCAGCTGCCGGACC	1563-1590
HBx-MT5-a	GGTCCGGCAGCTGCGAAGGCACAGACG	1563-1590
HBx-MT6-s	CTCATCTGCCGCAGCGGCTGCAGCTGCCGCCACC	1572-1606
HBx-MT6-a	GGTGGCGGCAGCTGCAGCCGCTGCGGCAGATGAG	1572-1606
HBx-MT7-s	GTGCACTTCGCTTCGCCGCTGCAGCTGCCGCGGCGGCCACC	1593-1625

HBx-MT7-a	GGTGGCCGCCGCGGCAGCTGCAGCGGCGAA GCGAAGTGCAC	1593-1625
HBx-MT8-s	CGCATGGAGGCCGCCGCGGCCGCCGCCGCG GTCTTG	1614-1643
HBx-MT8-a	CAAGACCGCGGCGGCGGCCGCGGCGGCCTC CATGCG	1614-1643
HBx-MT9-s	CCGTGAACGCCCACCAGGCCGCGGCCAAGG TCTTACATAAGAG	1635-1678
HBx-MT9-a	CTCTTATGTAAGACCTTGGCCGCGGCCTGGT GGGCGTTCACGG	1635-1678
HBx-MT10-s	ACGCCCACCAGGTCTTGCCCGCGGCCGCAC ATAAGAGGACT	1644-1685
HBx-MT10-a	AGTCCTCTTATGTGCGGCCGCGGGCAAGAC CTGGTGGGCGT	1644-1685
HBx-MT11-s	TCTTAGCTGCGGCGACTCTTG	1657-1678
HBx-MT11-a	AGTCGCCGCAGCTAAGACCTT	1657-1678
HBx-MT12-s	CCCAAGGTCTTACATAAGAGGGCTGCTGCAC TCTCAGCAATGTCAACGAC	1666-1716
HBx-MT12-a	GTCGTTGACATTGCTGAGAGTGCAGCAGCC CTCTTATGTAAGACCTTGGG	1666-1716
HBx-MT13-s	TACATAAGAGGACTCTTGGAGCCGCAGCAAT GTCAACGACCGAC	1675-1719
HBx-MT13-a	GTCGGTCGTTGACATTGCTGCGGCTCCAAGA GTCCTCTTATGTA	1675-1719
HBx-MT14-s	GACTCTTGGACTCTCAGCAGCGGCAGCGAC	1684-1727

	CGACCTTGAGGCA	
HBx-MT14-a	TGCCTCAAGGTCGGTCGCTGCCGCTGCTGA	1684-1727
	GAGTCCAAGAGTC	
HBx-MT15-s	GGACTCTCAGCAATGTCAACGGCCGCCGCT	1693-1743
	GAGGCATACTTCAAAGACTG	
HBx-MT15-a	CAGTCTTTGAAGTATGCCTCAGCGGCGGCCG	1693-1743
	TTGACATTGCTGAGAGTCC	
HBx-MT16-s	ATGTCAACGACCGACCTTGCGGCAGCCTTC	1702-1747
	AAAGACTGTGTGTTT	
HBx-MT16-a	AAACACACAGTCTTTGAAGGCTGCCGCAAG	1702-1747
	GTCGGTCGTTGACAT	
HBx-MT17-s	GCATACGCCGCAGCCTGTGTGTTTA	1711-1765
HBx-MT17-a	GTCTTTAAACACACAGGCTGCGGCGTAT	1711-1765
HBx-MT18-s	ACCTTGAGGCATACTTCAAAGACGCTGCGG	1720-1774
	CTAAAGACTGGGAGGAGTTGGGGG	
HBx-MT18-a	CCCCCAACTCCTCCCAGTCTTTAGCCGCAGC	1720-1774
	GTCTTTGAAGTATGCCTCAAGGT	
HBx-MT19-s	CTCCCCCAACTCCTCCGCGGCTGCAAACAC	1729-1784
	ACAGTCTTTGAAGTATGCCTCAAGG	
HBx-MT19-a	CCTTGAGGCATACTTCAAAGACTGTGTGTTT	1729-1784
	GCAGCCGCGGAGGAGTTGGGGGAG	
HBx-MT20-s	GACTGTGTGTTTAAAGACTGGGCGGCGGCG	1738-1789
	GGGGAGGAGATTAGGTTAAAG	
HBx-MT20-a	CTTTAACCTAATCTCCTCCCCCGCCGCCGCC	1738-1789
	CAGTCTTTAAACACACAGTC	

HBx-MT21-s	GAGTTGGCGGCGGCGATTAGGTTAAAG	1747-1774
HBx-MT21-a	CTTTAACCTAATCGCCGCCGCCAAC	1747-1774
HBx-MT22-s	GAGGAGGCTGCGGCAAAGGTCTTTGTAC	1756-1782
HBx-MT22-a	CAAAGACCTTTGCCGCAGCCTCCTCC	1756-1782
HBx-MT23-s	ATTAGGTTAGCGGCCGCTGTA	1765-1786
HBx-MT23-a	CTAGTACAGCGGCCGCTAACCTAATC	1765-1786
HBx-MT24-s	TCTTTGCAGCAGCAGGCTGTAG	1774-1796
HBx-MT24-a	TACAGCCTGCTGCTGCAAAG	1774-1796
HBx-MT25-s	CTAGGAGCCGCTGCGCATAAATTG	1783-1807
HBx-MT25-a	CAATTTATGCGCAGCGGCTCCTAG	1783-1807
HBx-MT26-s	CTGTAGGGCTGCAGCGGTCTGTTCAC	1792-1818
HBx-MT26-a	TGAACAGACCGCTGCAGCCCTACAG	1792-1818
HBx-MT27-s	TAAATTGGCCGCTGCACCAGCACC	1801-1824
HBx-MT27-a	GCTGGTGCAGCGGCCAATTTATG	1801-1824
HBx-MT28-s	GTTCAGCAGCAGCATGCAACTTT	1810-1829
HBx-MT28-a	GTTGCATGCTGCTGCTGAA	1810-1829
HBx-MT29-s	ACCAGCCGCCGCTTTCACCT	1819-1840
HBx-MT29-a	TGAAAGCGGCGGCTGGTGCTG	1819-1840
HBx-MT30-s	GCAACTTTGCCGCCGCTGCCTAATCA	1828-1836
HBx-MT30-a	GATTAGGCAGCGGCGGCAAAGTT	1828-1836
HBxStop-s	GCTAGGGTGTGCTGCTAACTGGATCCTCTAG	1387-1418
HBxStop-a	CTAGAGGATCCAGTTAGCAGCACACCCTAGC	1387-1418

HBV DNA sequence numbering system is according to Galibert et al.[1]

Mutagenic sites were underlined.

[1]Galibert, F., Mandart, E., Fitoussi, F., Tiollais, P., and Charnay, P. (1979) Nucleotide sequence of the hepatitis B virus genome (subtype ayw) cloned in *E. coli*. Nature 281, 646–650.

Supplementary Figure 1.

Mutagenesis of HBx.

The wild type amino acid sequence of the HBx C-terminal domain (A. aa positions 51-110; B. aa positions 101-154) is shown in the box. Each designated mutant was created by substituting consecutive residues by alanine. Unchanged amino acid residues were denoted by hyphens.

A. Hepatitis B X protein C-terminal domain (MT1-15; aa 51-110)

```
        51         61         71         81         91         101
WT HBx  AHLSLRGLPV CAFSSAGPCA LRFTSARRME ATVNAHQVLP KVLHKRTLGL SAMSTTDLEA
MT1     -AAA------ ---------- ---------- ---------- ---------- ----------
MT2     ----AAA--- ---------- ---------- ---------- ---------- ----------
MT3     -------AAA ---------- ---------- ---------- ---------- ----------
MT4     ---------- AAA------- ---------- ---------- ---------- ----------
MT5     ---------- ---AAA---- ---------- ---------- ---------- ----------
MT6     ---------- ------AAAA AAA------- ---------- ---------- ----------
MT7     ---------- ---------- ---AAAAAAA ---------- ---------- ----------
MT8     ---------- ---------- ---------- AAAAAAA--- ---------- ----------
MT9     ---------- ---------- ---------- -------AAA ---------- ----------
MT10    ---------- ---------- ---------- ---------- AAA------- ----------
MT11    ---------- ---------- ---------- ---------- ---AAA---- ----------
MT12    ---------- ---------- ---------- ---------- ------AAA- ----------
MT13    ---------- ---------- ---------- ---------- ---------A AA--------
MT14    ---------- ---------- ---------- ---------- ---------- --AAA-----
MT15    ---------- ---------- ---------- ---------- ---------- -----AAA--
```

B. Hepatitis B X protein C-terminal domain (MT16-30; aa 101-154)

```
        101        111        121        131        141        151  154
WT HBx  SAMSTTDLEA YFKDCVFKDW EELGEEIRLK VFVLGGCRHK LVCSPAPCNF FTSA*
MT16    --------AA A--------- ---------- ---------- ---------- ----*
MT17    ---------- -AAA------ ---------- ---------- ---------- ----*
MT18    ---------- ----AAA--- ---------- ---------- ---------- ----*
MT19    ---------- -------AAA ---------- ---------- ---------- ----*
MT20    ---------- ---------- AAA------- ---------- ---------- ----*
MT21    ---------- ---------- ---AAA---- ---------- ---------- ----*
MT22    ---------- ---------- ------AAA- ---------- ---------- ----*
MT23    ---------- ---------- ---------A AA-------- ---------- ----*
MT24    ---------- ---------- ---------- --AAA----- ---------- ----*
MT25    ---------- ---------- ---------- -----AAA-- ---------- ----*
MT26    ---------- ---------- ---------- --------AA A--------- ----*
MT27    ---------- ---------- ---------- ---------- -AAA------ ----*
MT28    ---------- ---------- ---------- ---------- ----AAA--- ----*
MT29    ---------- ---------- ---------- ---------- -------AAA ----*
MT30    ---------- ---------- ---------- ---------- ---------- AAA-*
```

Supplementary Figure 2

Agarose gel electrophoresis analysis of circularized, plasmid-free HBV DNA used for HBV transfection. From a cloned plasmid, a single genome length HBV DNA, free of any vector DNA, was released by restriction digestion. Following self-ligation of the HBV DNA, a circularized HBV DNA (HBV3) of 3.2 kb was formed and was analyzed by gel electrophoreses. M: supercoiled DNA marker (New England Biolabs).

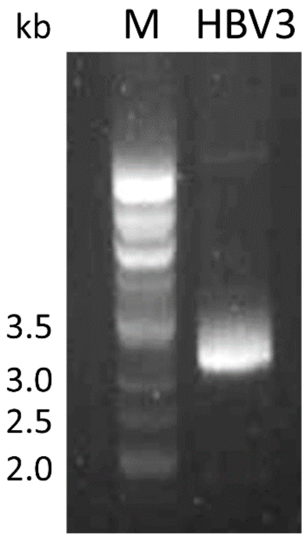

Supplement: Supplementary file 1 — Suppementary Materials. [file 41598_2020_57637_MOESM1_ESM.pdf]
